# Magnetic Response Detects the Strength of Carrageenan Network

**DOI:** 10.3390/gels8090584

**Published:** 2022-09-14

**Authors:** Masahiro Kaneko, Mika Kawai, Tetsu Mitsumata

**Affiliations:** Graduate School of Science and Technology, Niigata University, Niigata 950-2181, Japan

**Keywords:** magnetic gel, stimuli-responsive gel, soft material, magnetorheological effect

## Abstract

The effect of carrageenan concentration on the magneto-rheological effect of magnetic gels with a magnetic particle concentration of 50 wt.% was investigated under a magnetic field of 50 mT by dynamic viscoelastic measurements. The change in the storage modulus for magnetic gels due to the magnetic field was 3.0 × 10^3^ Pa at a carrageenan concentration of 1.0 wt.% and increased with the concentration. The modulus change showed a maximum of 2.3 × 10^4^ Pa at ~2.0 wt.% and became lower at higher concentrations. This is an interesting phenomenon, which was first observed in this study. The critical strain, the strain where the storage modulus intersects the loss modulus in the strain dependence of the modulus, was much higher than that for carrageenan gels, indicating a strong interaction between the magnetic particles and carrageenan. At 0 mT, the critical strain for the magnetic gels increased remarkably with decreasing the concentration, indicating that magnetic gels have a structure that does not flow easily at concentrations below 1.75 wt.%. It is considered that the structure hardly flows, hindering the movement of particles. At high concentrations, the SEM photographs showed both a particle network of magnetic particles and a dense carrageenan network. It can be considered that the movement of magnetic particles was hindered due to these factors at high concentrations.

## 1. Introduction

Magnetic gels are stimuli-responsive soft materials [1,2,3,4,5] and their physical properties alter in response to magnetic fields. The magnetic response for magnetic soft materials is, in general, drastic; therefore the material has attracted considerable attention as actuators in the next generation [6,7,8,9,10,11,12,13]. When a magnetic field is applied to magnetic gels, the magnetic particles contact each other and form a chain-like structure. As a result, the elastic modulus of the magnetic gel becomes higher than that without a magnetic field. This is called the magneto-rheological effect (MR effect).

Since the magnetic particle is physically bound to the polymer network, it receives a resistance force from the polymer network when moving in the gel, which causes a reduction in the magnitude of the MR effect. In other words, the chain structure of magnetic particles is created less when the magnetic particles are difficult to move in the gel, resulting in the reduction in the MR effect. Hence, the amplitude of the MR effect tells us the nature of the polymer network, and that magnetic particles are difficult or easy to move. The amplitude of the MR effect increases with a decrease in the elastic modulus of the matrix for magnetic gels. In addition, magnetic gels with a matrix of natural polymers such as carrageenan gels and agar gels [11,12,14] show a larger change in elastic modulus than those with synthetic polymers. This suggests that the network of natural polymers is an easy field of movement for magnetic particles. We consider that this feature is due to the cross-linking points of natural polymers. Since the cross-linking points of natural polymer gels are formed by hydrogen (physical) bonds, it is considered that the cross-linking points are broken when magnetic particles move, when a magnetic field is applied.

Most of our previous experiments, thus far, were performed at a concentration of magnetic particles with 70 wt.% and magnetic field strength of 500 mT. At high concentrations, magnetic particles are able to contact each other, even at low magnetic fields. Therefore, the resistant force from the polymer network is not observable at all. In fact, the change in storage modulus monotonously decreased with the carrageenan concentration at a carrageenan concentration of 70 wt.% and 500 mT, which is a simple result where the softer the gel, the larger the modulus change [15]. The change in storage modulus at 50 wt.% and 50 mT was also investigated for another objective (determination of the diameter of secondary particles) [16]. The changes in the storage modulus at 40 wt.% and 50 wt.% were ~8.6 × 10^3^ Pa and ~1.8 × 10^4^ Pa for carrageenan gel with a carrageenan concentration of 1.0 wt.%, respectively. This result indicates that an experimental condition of 50 wt.% and 50 mT is suitable for the present study to determine the change in modulus while varying the carrageenan concentration. Similarly, at high magnetic fields, the magnetic forces acting on the magnetic particles are too strong, and the resistive forces due to the polymer network cannot be observed. Accordingly, experimental conditions at both low concentrations and low magnetic fields are suitable for measuring the resistant force acting on magnetic particles from the polymer network. In this study, we investigate the effect of the magnetic field on the complex modulus of carrageenan gels at low concentrations of magnetic particles and at a low magnetic field and discuss the correlation between the amplitude of storage modulus and the viscoelasticity of the carrageenan network.

## 2. Results and Discussion

**Figure 1:**
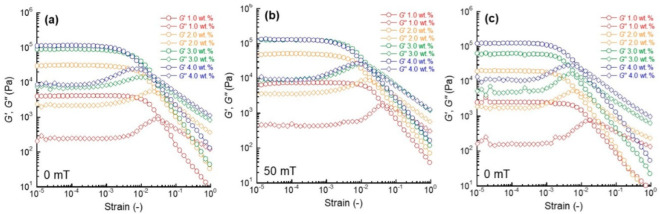
The strain dependence of the storage and loss moduli for magnetic gels with a concentration of 50 wt.% at (**a**) 0 mT and (**b**) 50 mT and for (**c**) the carrageenan gels without magnetic particles at 0 mT.

Figure 1a shows the strain dependence of the storage modulus *G*’ and loss modulus *G*” at 0 mT for carrageenan magnetic gels with various carrageenan concentrations. The *G*’ and *G*” for 1.0 wt.% carrageenan magnetic gel were constant at strains below 4.3 × 10^−3^ and 2.3 × 10^−3^, respectively. The *G*’ decreased rapidly above a strain of 4.3 × 10^−3^ while the *G*” peaked at a strain of 4.1 × 10^−2^, intersecting with the *G*’. A similar behavior was seen at other concentrations of the magnetic gels. Figure 1b shows the strain dependence of the *G*’ and *G*” at 50 mT for carrageenan magnetic gels with various carrageenan concentrations. The *G*’ and *G*” for the 1.0 wt.% carrageenan magnetic gel were constant at strains below 5.4 × 10^−3^ and 1.7 × 10^−3^, respectively. The *G*’ decreased significantly above a strain of 5.4 × 10^−3^, while the *G*” peaked at a strain of 4.8 × 10^−2^, crossing the *G*’. At all concentrations, a similar behavior was seen at other concentrations of the magnetic gels. Figure 1c shows the strain dependence of the *G*’ and *G*” at 0 mT for the carrageenan gels without magnetic particles. The *G*’ and *G*” for the 1.0 wt.% carrageenan gel was constant at strains below 2.4 × 10^−3^ and 1.3 × 10^−3^, respectively. The *G*’ dropped largely to a low value at a strain of 2.4 × 10^−3^, while the *G*” peaked at a strain of 1.8 × 10^−2^ and intersected the *G*’. At all concentrations, a similar behavior was seen at other concentrations of the carrageenan gels.

**Figure 2:**
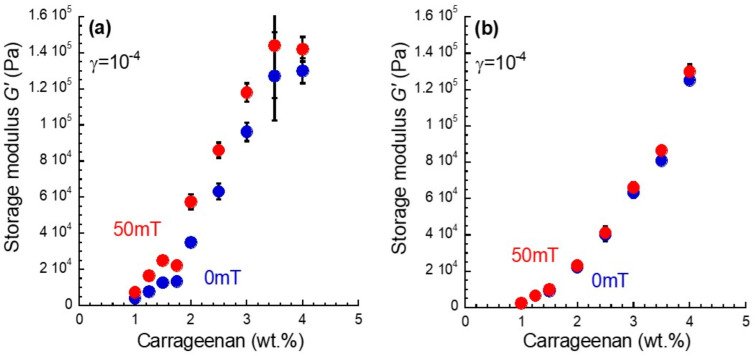
The carrageenan concentration dependence of the storage moduli at 0 mT and 50 mT for (**a**) magnetic gels with a concentration of 50 wt.% and (**b**) carrageenan gels without magnetic particles.

Figure 2a shows the relationship between the storage modulus in the linear viscoelastic regime and carrageenan concentration for magnetic gels. The storage modulus at 0 mT increased with the concentration. It should be noted that the storage modulus increased largely at a concentration of 2.0 wt.%. The slope of the storage modulus against the concentration also changed significantly around this concentration. This strongly indicates that the structure of the carrageenan network and/or the dispersibility of magnetic particles dramatically changed at this concentration. It was found that the storage modulus at 50 mT also increased largely at a concentration of 2.0 wt.%. Figure 2b shows the relationship between the storage modulus in the linear viscoelastic regime and the carrageenan concentration for carrageenan gels without magnetic particles. Both storage moduli at 0 mT and 50 mT increased with the concentration. It is clear that there was no magnetic field effect on the storage modulus for the magnetic gels. At concentrations below 1.5 wt.%, the storage modulus for the carrageenan gels equaled those for the magnetic gel at 0 mT. This indicates that the filling effect of magnetic particles on the bulk modulus was less at this concentration range.

**Figure 3:**
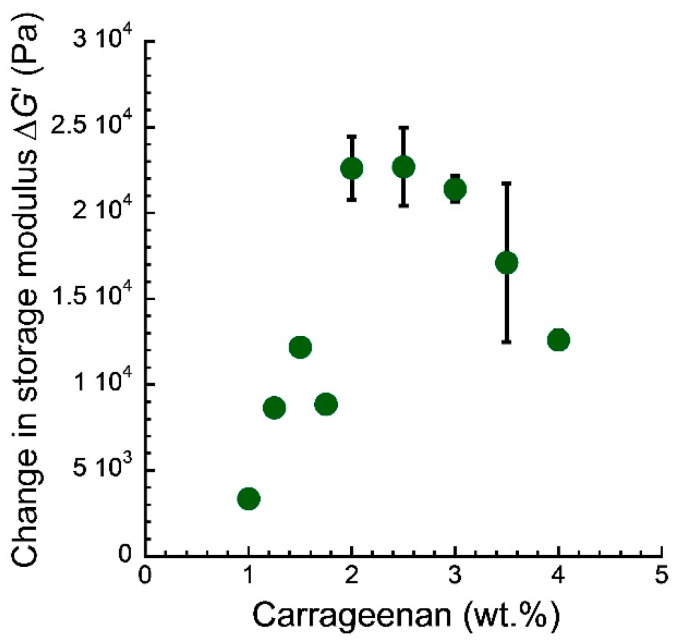
The relationship between the change in storage modulus and carrageenan concentration for magnetic gels with a concentration of 50 wt.%.

Figure 3 indicates the change in storage modulus Δ*G*’ and carrageenan concentration for the carrageenan magnetic gels; Δ*G*’ is the difference between the storage moduli at 0 mT, *G*’_0_, and 50 mT, *G’*_50_, (Δ*G’* = *G’*_50_ − *G’*_0_). The Δ*G’* for the magnetic gel was 3.0 × 10^3^ Pa at 1.0 wt.% and increased with the concentration at concentrations below 2.0 wt.%. The Δ*G’* showed a maximum of 2.3 × 10^4^ Pa at ~2.0 wt.% and decreased to a low value of 1.3 × 10^4^ Pa at 4.0 wt.%. This is an interesting phenomenon, which was first observed in this study. In magnetic soft materials, the Δ*G’* increases with a decrease in *G*’_0_ since the chain structure of magnetic particles develops as there is an increase in the mobility of magnetic particles. Accordingly, the Δ*G*’ should increase simply with a decrease in the carrageenan concentration. There should be a mechanism to reduce the Δ*G*’, for example, a decrease in the chain density due to the formation of secondary particles [14], otherwise chains were not created at all. Carbonyl iron is relatively unlikely to create secondary particles in carrageenan aqueous solutions. This is because carrageenan chains adsorb on the surface of carbonyl iron and electrostatically repulse between the particles [14,17]. It is also unlikely that chain structures were not formed. Actually, a large Δ*G*’ was found at high concentrations; a dispersion that was the same magnetic particle in pure water at the same concentration demonstrated the storage moduli of 4.2 × 10^1^ Pa at 0 mT and 3.4 × 10^4^ Pa at 50 mT (i.e., Δ*G*’ = 3.4 × 10^4^ Pa). Therefore, it can be considered that this phenomenon reflects the viscoelastic property of the carrageenan network. A magnetic field of 50 mT is almost the lower limit of the field that can measure the change in storage modulus accurately (~10^4^ Pa order) [14]. The concentration of 50 wt.% is just above the percolation threshold of chain formation, which is also almost the lower limit of the concentration that can measure the modulus accurately [16]. Thus, we observed a balanced state of magnetic interaction and network elasticity at the optimized condition.

**Figure 4:**
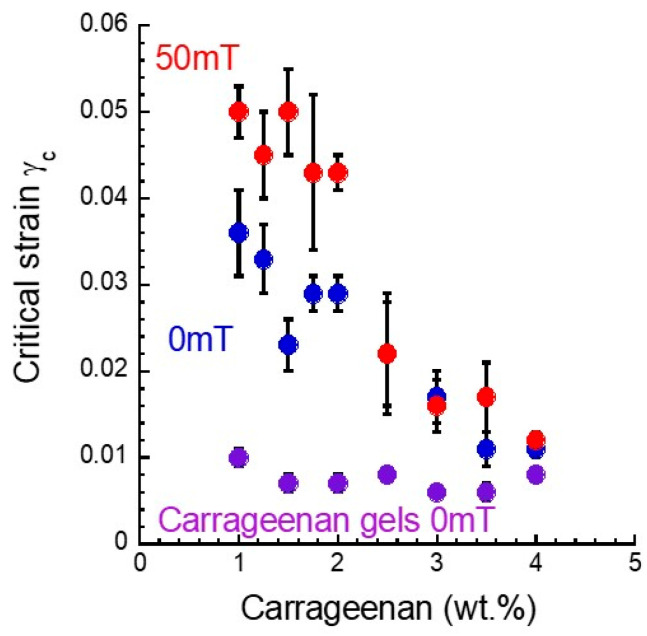
The relationship between the critical strain and carrageenan concentration for magnetic gels with a concentration of 50 wt.% and carrageenan gels without magnetic particles.

Figure 4 shows the relationship between the critical strain and carrageenan concentration for magnetic gels and carrageenan gels without magnetic particles. The critical strain is a yield point intersecting the *G*’ and *G*” in Figure 1, which is the onset of a fluid-like response and has been related to the failure of the network structure [18,19,20]. For both magnetic and carrageenan gels, the critical strain was distributed in a strain region of 10^−3^~10^−1^.

The critical strain of carrageenan gels without magnetic particles was distributed around 0.008, which was independent of the concentration, meaning that the strength of the carrageenan network was almost constant. For magnetic gels at 0 mT, the critical strain was higher than that of the carrageenan gel, suggesting that the carrageenan network was reinforced by carbonyl iron particles. The critical strain at a concentration of 4.0 wt.% was 0.01 and increased with a decrease in the concentration. This strongly indicates that the carrageenan network is tough at lower concentrations, which is hard to flow in shear stress. It can be considered that the decrease in Δ*G*’ seen at low concentrations is due to a high yield stress by which the mobility of magnetic particles was lost. The critical strain at 50 mT was far higher than that at 0 mT at concentrations below 2.0 wt.% (~0.05), suggesting that a rigid structure with low fluidity was produced. Generally, the linear viscoelastic regime is widened in the presence of a magnetic field compared to that in the absence of a magnetic field, which agrees with the high critical strain observed here. Above 2.5 wt.%, no clear change in the critical strain was observed, indicating that the carrageenan network and/or the particle structure was not changed in this concentration range.

**Figure 5:**
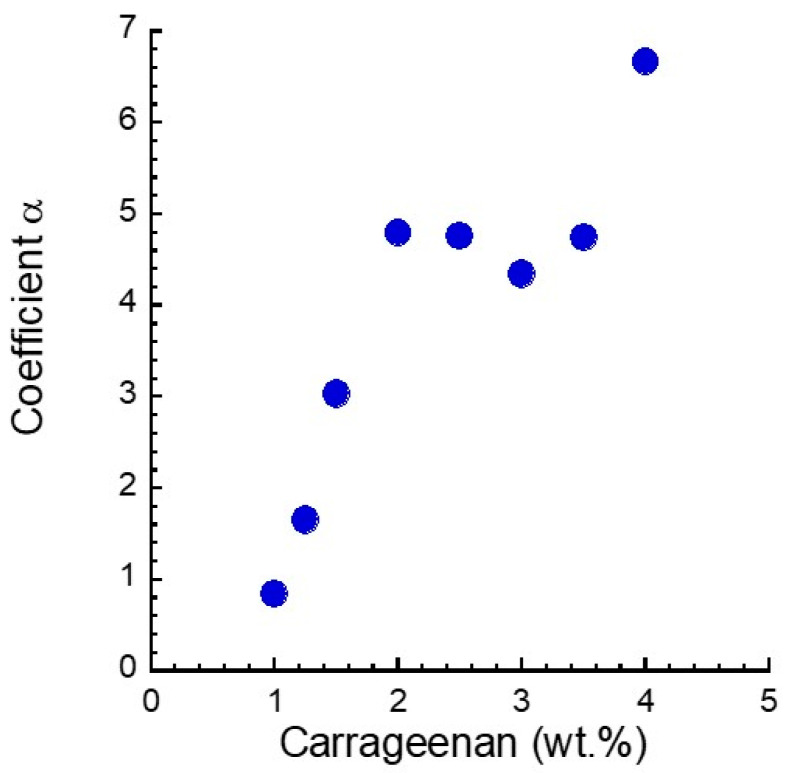
The coefficient in Equation (1) against the carrageenan concentration for magnetic gels with a concentration of 50 wt.%.

At concentrations above 2.5 wt%, the storage modulus took a high value that was relative to the modulus at concentrations below 2.0 wt%, as shown in Figure 2. The storage modulus at 0 mT for the magnetic gel *G*’ can be written by the following Einstein equation [21].

(1) $$G^{\prime }={G^{\prime }}_{\mathrm{matrix}}\left(1+\alpha \phi \right)$$

where *G’*_matrix_ is the storage modulus of the matrix at 0 mT and *ϕ* is the volume fraction of the magnetic particles (=0.12). The *α* is a parameter relating to the energy loss for the rotation of fillers. Figure 5 exhibits the relationship between the parameter *α* in Equation (1) and the carrageenan concentration for magnetic gels, assuming that the *G*’_matrix_ equals the storage modulus for carrageenan gels without magnetic particles in Figure 2b. The value of *α* for the magnetic gels at concentrations below 1.25 wt.% was extremely low. This indicates that there was no interaction between the magnetic particles and carrageenan gel. However, this idea is clearly incorrect; the critical strain revealed that the magnetic gels had a tough structure at the concentration range that was not destroyed by large shear. Accordingly, it was considered that the storage modulus of the carrageenan gel *G*’_matrix_ in Equation (1) was overestimated (i.e., the *G*’_matrix_ was reduced by mixing with magnetic particles). Actually, we have clarified that carrageenan chains were adsorbed on the surface of the carbonyl iron particles in its aqueous solution [17]. At concentrations above 1.5 wt.%, the value of *α* was far higher than 2.5, indicating that secondary particles or a particle network was produced in the magnetic gels.

**Figure 6:**
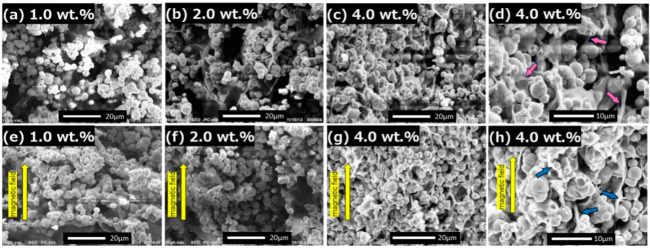
The SEM photographs for magnetic gels with various carrageenan concentrations at 0 mT (**top**) and 50 mT (**bottom**). Magnetic particle concentration: 50 wt.%.

Figure 6 displays the scanning electron microphotographs for the magnetic gels in the absence and presence of a magnetic field of 50 mT. At 0 mT, strings or membranes of carrageenan gel were observed to be increased with an increase in the carrageenan concentration. It was also seen at a concentration of 4.0 wt.% that spaces between the magnetic particles were fully filled with carrageenan gel, and the magnetic particles seemed to be interconnected. In addition, many strings of carrageenan were observed to interconnect between the magnetic particles, as indicated by red and blue arrows. This might be a factor by which the mobility of magnetic particles is reduced. Both the dense network of carrageenan and the interconnection effect might affect the decrease in Δ*G*’ for the magnetic gels at concentrations above 2.5 wt.%. At 50 mT, no clear chain structure of the magnetic particles was seen at all concentrations. Two main results, a clear increase in the storage modulus and high critical strain, indicate the existence of chain structures. Therefore, it can be considered that an anisotropic structure of magnetic particles can be formed at 50 mT, however, it did not develop into a chain structure that could be visualized in the SEM photographs.

## 3. Conclusions

The effect of carrageenan concentration on the MR effect of magnetic gels was investigated by dynamic viscoelastic measurement at low concentrations of magnetic particles and at a low magnetic field. When the magnetic field strength is high, the change in storage modulus is simply inversely proportional to the modulus of the gel. This means that the mechanical properties of the network cannot be found from the amplitude of the magneto-rheological effect. When the concentration of magnetic particles is low or the magnetic field is low, the modulus change is small, leading to reduced accuracy. The experimental conditions in this study are optimal for detecting the mechanical properties of the gel network and ensuring the accuracy of the modulus change. It was first observed that the change in the storage modulus by the magnetic field was found to show a maximum. At low concentrations below a concentration of 2.0 wt.%, it is considered that the reduction in the MR effect is due to a tough network of carrageenan that can endure high strain. At high concentrations above a concentration of 2.5 wt.%, the reduction in the MR effect is caused by both the effects of the appearance of a particle network and dense network of carrageenan. Thus, the strength of the polymer network can be detected by the magnetic response. The features obtained here also provide an insight into the movement of solids in soft materials, which would lead to the development of devices enabling non-contact measurement of the local elasticity in vivo.

## 4. Materials and Methods

### 4.1. Preparation of Magnetic Gels

κ-Carrageenan (*M*_w_ = 857 kDa, CS-530, San-Ei Gen F.F.I., Osaka, Japan) was used as a matrix of polysaccharides. The carrageenan was dissolved in pure water at 100 °C for 3 h to prepare the aqueous solutions with concentrations of 1.0~4.0 wt.%. Carbonyl iron (CS Grade BASF SE., Ludwigshafen am Rhein, Germany) with a diameter of 7.0 µm was used as the magnetic particle. The carbonyl iron particles were dispersed in the carrageenan aqueous solutions at 100 °C and then mixed using a mechanical mixer to obtain the pre-gel solutions. The concentration of magnetic particle was constant at 50 wt.%, which corresponded to a volume fraction *ϕ* of 0.12. The pre-gel solution was poured into a mold made of a silicone spacer and glass plates at room temperature. The gel was then stored in a refrigerator at 4 °C for 1 h. The sample was 1 mm in thick and 20 mm in diameter. Carrageenan gels without magnetic particles were also prepared in a similar manner with magnetic gels. The diameter of the carbonyl iron was determined to be 7.4 ± 0.2 µm by a particle size analyzer (SALD-7000, Shimadzu Co. Ltd., Kyoto, Japan). The saturation magnetization was measured as 218 emu/g by a SQUID magnetometer (MPMS, Quantum Design Inc., San Diego, CA, USA).

### 4.2. Dynamic Viscoelastic Measurement

The dynamic viscoelastic measurement for the magnetic gels was carried out at a temperature of 20 °C and a frequency of 1 Hz using a rheometer (MCR301, Anton Par Pty Ltd., Graz, Austria) with an electromagnetic system (PS-MRD) and a non-magnetic parallel plate (PP20/MRD). The strain was varied from 10^−5^ to 1. The normal force initially applied was approximately 0.3 N. The magnetic field strength at the sample stage in the rheometer was measured using a Gauss meter (TM-601, Kanetec Co., Ltd., Nagano, Japan). The magnetic field strength was 50 mT when the excitation current was 0.292 A. The mean values and standard errors of the storage and loss moduli for three different samples from one batch were evaluated.

### 4.3. SEM Observations

To clarify the morphologies of the magnetic particles and carrageenan network, a cross-section of the dried magnetic gels was observed using a scanning electron microscope (SEM) (JCM-6000 Neoscope, JEOL Ltd., Tokyo, Japan) at an acceleration voltage of 15 kV. The dried magnetic gels were obtained by freeze-drying for 10 h under a magnetic field of 50 mT using a permanent magnet.
